# Social Connectedness Buffering the Interaction Between Problematic Internet Use and Psychopathological Symptoms Among Adolescents: Undirected and Bayesian Network Analyses

**DOI:** 10.2196/92473

**Published:** 2026-08-31

**Authors:** Jiaqi Xu, Wenxin Bao, Kexin Zhou, Qiyuan Cao, Min Hu, Jing Li, Xiacan Chen, Jiajun Xu

**Affiliations:** 1 Mental Health Center, West China Hospital, Sichuan University, No. 28, Telecom South Street, Chengdu, Sichuan, 610000, China, 86 17381883373; 2 Psychiatry department, Chengdu Southwest Rehabilitation Hospital, Chengdu, China; 3 Institute of Forensic Medicine, West China School of Basic Medical Sciences and Forensic Medicine, Sichuan University, Chengdu, China; 4 Département de psychiatrie et addictologie, Université de Montréal Centre de Recherche Institut National de Psychiatrie Légale Philippe-Pinel, Montreal, QC, Canada

**Keywords:** problematic internet use, psychopathological symptoms, social connectedness, network analysis, directed acyclic graph

## Abstract

**Background:**

Problematic internet use (PIU) in adolescents often co-occurs with psychopathological symptoms. Social connectedness (SC) is a potential protective factor for both PIU and psychopathological symptoms, but how SC interacts with the PIU-psychopathology system remains unclear.

**Objective:**

This study aimed to characterize the factor-level comorbidity network linking PIU with psychopathological symptoms among adolescents at high risk of PIU and to clarify how offline and online SC function in this system. We also applied Bayesian network structure learning to explore algorithm-preferred edge orientations.

**Methods:**

This multicenter study included 9407 adolescents aged 12 to 18 years from 4 provinces in China. PIU, depression, anxiety, irritability, repetitive thoughts and behaviors, anger, aggression, and SC were assessed. We estimated an undirected factor-level symptom network and a directed acyclic graph derived from a Bayesian network structure to characterize associations and conditional dependency structures among SC, PIU, and 8 psychopathological symptoms.

**Results:**

The integrative symptom network encompassing PIU, internalizing and externalizing symptoms, and SC showed great stability and accuracy. Anxiety, anger, and depression were identified as central bridging symptoms that reinforce the interplay between PIU and other psychopathological symptoms. Offline SC exhibited the highest number of negative edges and the highest bridge expected influence value, followed by online SC. In the exploratory directed acyclic graph, offline SC showed algorithm-selected outgoing arrows to PIU salience and to several internalizing and externalizing symptoms, including depression, anxiety, verbal aggression, and hostility.

**Conclusions:**

PIU and psychopathological symptoms formed a closely interconnected factor-level network among adolescents in the high-risk PIU group. Offline SC occupied a prominent negative bridge position, whereas online SC showed a secondary negative network position and was positively connected with offline SC. These findings support further evaluation of offline SC as a candidate prevention and intervention target.

## Introduction

Problematic internet use (PIU), characterized by excessive internet use that causes distress or daily function impairment [1], has emerged as an increasingly serious public health concern. Recent evidence indicates that its prevalence ranges from roughly 20% to 44.6% across different regions of the world [2]. Adolescents with PIU are at increased risk for multidomain adverse outcomes [3-5] and often have co-occurring psychopathological symptoms [6-10]. These symptoms can be categorized as internalizing problems, which reflect inwardly directed emotional distress, such as depression, anxiety, and repetitive thoughts and behaviors, and externalizing problems, which involve outwardly expressed emotional or behavioral dysregulation, such as irritability, anger, aggression, and hostility [11]. Emotional and behavioral difficulties may contribute to excessive internet use [12,13], while PIU may also intensify preexisting psychological symptoms [14,15]. However, the interactive pattern between PIU and psychopathological symptoms remains relatively underexplored, such as which specific psychopathological dimensions are most tightly coupled with PIU and whether certain symptoms serve as key bridges linking the 2 domains.

The network approach conceptualizes psychopathology as a system of interacting symptoms or factors, represented as nodes connected by statistical edges [16,17]. It has been increasingly applied to characterize the complex interplays among comorbid psychopathological symptoms, including PIU and other mental health problems in adolescents and young adults [18-21]. Previous studies have identified bridge features such as gaming for escape or mood relief in networks of internet gaming disorder and depression [22]. Using a longitudinal cross-lagged panel network, Zhou et al [21] further reported gender-specific symptom pathways, with depressed mood serving as a bridge symptom for boys and irritability for girls. However, existing large-scale PIU network studies have mainly relied on broad community samples, including college students and mixed adolescent-young adult populations [20,23]. Conversely, clinically recruited network studies often focused on disorder-specific populations (eg, adolescents with major psychiatric disorders [18]) and treated PIU primarily as a comorbid condition. Large-scale symptom-level investigations have not yet been examined in nonclinical adolescent samples presenting with high-risk PIU symptoms. This gap may bias network estimates: community samples may underrepresent severe PIU, whereas clinical samples may be shaped by the primary disorder and treatment condition.

Furthermore, most PIU network studies have used undirected models [23-25], which characterize conditional associations but do not provide information about edge orientation. Bayesian network structure learning can be used to estimate directed acyclic graphs (DAGs), in which some edges are assigned algorithm-preferred orientations based on patterns of conditional dependence and model fit [26,27]. DAGs can complement undirected networks by providing an exploratory representation of possible directional relationships [28] and generating hypotheses for future longitudinal or interventional studies [29].

Beyond the interplay among symptoms, identifying modifiable protective factors that can influence these relationships is equally important for intervention. Social connectedness (SC) refers to a subjective sense of belonging, closeness, and integration within one’s social relationships [30]. SC is closely related to social support, as both constructs concern individuals’ social relationships. However, we focus on SC because it captures a broader, generalized sense of relational belonging. Among adolescents, stronger SC is linked to lower levels of PIU [31,32] and fewer depressive and anxiety symptoms [33,34]. Our previous studies also found that SC statistically mediated the association between emotional symptoms and PIU [35,36]. Together, these findings suggest that SC may occupy an important position across PIU and psychopathology, although it has rarely been incorporated into network models or DAGs of these domains.

SC may also differ across offline and online contexts. Offline SC reflects a sense of belonging and closeness in face-to-face relationships and has generally been associated with better emotional well-being [37,38]. In contrast, online SC, which develops through digitally mediated interactions, has shown mixed associations with PIU and mental health. Moderate online social engagement may provide social support [39], whereas excessive reliance may heighten PIU and emotional distress [40]. To distinguish these contexts, we adapted parallel offline and online versions of the Social Connectedness Scale-Revised (SCS-R) by adding the qualifiers “in the real world” and “in the online world,” respectively. This contextualization enabled a direct comparison of how offline and online SC are positioned within the same PIU-psychopathology network. As these adapted versions had not been independently psychometrically validated before the study, the present analysis provides an initial examination of context-specific SC. It remains unclear how offline and online SC are differentially related to the PIU-psychopathology network. Addressing this gap may help identify actionable early targets for prevention.

Against this background, this study aimed to characterize the factor-level network linking PIU, psychopathological symptoms, and online and offline SC among adolescents with high-risk PIU symptoms. We hypothesized that PIU factors would be positively associated with both internalizing and externalizing symptoms (IES), whereas SC, particularly offline SC, would show predominantly negative associations with these domains and occupy a prominent negative bridge position in the network. We additionally applied DAG analysis as an exploratory approach to characterize algorithm-preferred edge orientations and generate directional hypotheses for future longitudinal and interventional research.

## Methods

### Study Design and Participants

This was a large-scale, cross-sectional study conducted from September 2022 to March 2023 in various regions across China, including Jiangsu in the east, Fujian in the south, Sichuan in the west, and Xinjiang in the north. The 4 provinces were selected to increase geographic diversity. Within each province, 1 or 2 middle schools were recruited through convenience sampling. Class-based cluster sampling was then conducted within participating schools, and all eligible students aged 12 to 18 years in the selected classes were invited to participate. A total of 11,946 participants completed an anonymous, self-reported online questionnaire via the Chinese survey platform wjx.cn during a designated computer class under the supervision of their teachers. The flowchart of the procedure was listed in our previous study [35].

For data quality assurance, 2539 questionnaires were excluded based on the following criteria: (1) participants who reported an age below 12 or above 18 years (n=509); (2) participants who completed the survey in less than 10 minutes (n=632); (3) participants who responded “false or entirely false” to the question “To what extent does your survey reflect the real situation? (options: entirely false, false, general, true, entirely true)” at the end of the survey (n=1231); (4) questionnaires with incomplete essential demographic information (n=139); and (5) questionnaires excluded due to duplication (n=28). In total, data from 9407 participants (mean age 14.90, SD 1.61 y) were included in the study, of whom only those with moderate to severe PIU (n=2055) were retained for the subsequent network analyses.

### Ethical Considerations

The study received ethical approval from the Ethics Committee of West China Hospital, Sichuan University (number 2019‐907). Written informed consent from the participants’ guardians and assent from the adolescents were obtained before data collection. Participation was voluntary, and all survey responses were anonymous.

### Measures

#### Young’s 20-Item Internet Addiction Test

We used Young’s 20-item Internet Addiction Test (IAT-20) [41,42], to assess symptoms of PIU among participants. This self-reported scale comprises 20 questions using a 5-point Likert scale. Based on prior research, factor analysis identified 6 structural factors: salience (preoccupation with the internet), excessive use, neglect work, anticipation, lack of control (has trouble managing online time), and neglect social life [43]. Notably, the item *How often do you form new relationships with fellow online users?* (which is categorized under the factor of neglect social life) exhibits considerable overlap with the variable of online SC in this study. Thus, we excluded the factor neglect social life (which also accounted for the least variance in factor analysis, as reported by Widyanto and McMurran [43]) from subsequent network analysis. Consistent with the validation of the Chinese IAT-20, participants with scores on the IAT-20 ≥50 were operationally classified into the high-risk PIU group [42,44,45]. The Cronbach α coefficients for the factors of salience, excessive use, neglect work, anticipation*,* and lack of control were 0.84, 0.81, 0.64, 0.63, and 0.79, respectively.

#### *Diagnostic and Statistical Manual of Mental Disorders* (Fifth Edition, Text Revision) Level-2 Scales

A set of *Diagnostic and Statistical Manual of Mental Disorders* (Fifth Edition, Text Revision; *DSM-5-TR*) Level-2 measures for children aged 11 to 17 years, developed by the American Psychiatric Association [46], was used to assess the severity of depression, anxiety, irritability, and repetitive thoughts and behaviors*.* Higher scores on each subscale indicated greater symptom severity. The Chinese version was translated and validated by Wang and Zhong [47]. The Cronbach α values for the subscales of depression, anxiety, irritability, and repetitive thoughts and behaviors were 0.97, 0.95, 0.92, and 0.93, respectively.

#### Buss-Perry Aggression Questionnaire

The Chinese version of the Buss-Perry Aggression Questionnaire was used for the assessment of aggression [48]. The questionnaire includes 29 items, each scored on a scale of 1 to 5. It assesses 4 distinct domains of aggression (physical aggression, verbal aggression, anger, and hostility), with higher scores indicating elevated levels of aggressive behavior [49,50]. The Cronbach α coefficients for the factors of physical aggression, verbal aggression, anger, and hostility were 0.79, 0.70, 0.80, and 0.84, respectively.

#### SCS-R

SCS-R, originally developed by Lee and Robbins [51], was used to assess SC. The scale comprises 20 items, each rated on a 6-point scale (1=strongly disagree, 6=strongly agree), with higher scores indicating stronger feelings of SC. To separately measure online SC (on_SC) and offline SC (off_SC), the original items were adapted by adding the phrases “in the online world” and “in the real world” preceding each item, respectively. Although the adapted scales had not been previously validated, we conducted an initial psychometric assessment. The Cronbach α coefficients for on_SC and off_SC were 0.76 and 0.90, respectively, indicating acceptable internal consistency.

### Data Analysis

#### Network Estimation

Network analyses were performed in the high-risk PIU group, defined as adolescents scoring ≥50 on the IAT-20 (n=2055). All analyses were conducted using R (version 4.2.3; R Foundation for Statistical Computing). The *qgraph* package was used to estimate and visualize networks, and glasso implemented graphical least absolute shrinkage and selection operator regularization. Average scores derived from each subscale were used as nodes in the network analysis. The Spearman correlations among IAT factors, psychopathology symptoms, Buss-Perry Aggression Questionnaire factors, and SCs were calculated based on the graphical Gaussian model. The edge weight parameters resulting from the graphical Gaussian model were regularized using least absolute shrinkage and selection operator and selected using the extended Bayesian information criterion with tuning parameter γ=0.50 [16,52]. Depression, anxiety, and repetitive thoughts and behaviors were conceptualized as internalizing psychopathological symptoms in this study, while irritability, verbal aggression, physical aggression, anger, and hostility were conceptualized as externalizing symptoms. The comorbidity network of PIU symptoms (factors generated from IAT-20) and 8 IES was first examined to identify the central symptoms and investigate potential bridge nodes. Subsequently, *on_SC* and *off_SC* were incorporated to further explore their impact on the comorbidity network.

This resulted in an integrative symptom network encompassing PIU, IES, and SC (PIU-IES-SC). The visualized networks represent each symptom or factor as a node, with an edge connecting 2 nodes indicating the presence of an association even after accounting for all other nodes in the same network [52]. The weight of an edge is indicated by its corresponding thickness and saturation. In addition, node predictability was assessed using the *mgm* package and presented as a ring chart surrounding each node, indicating the extent to which it shares variance with its neighboring nodes [17].

#### Accuracy and Stability

The *bootnet* package was used to assess edge weight accuracy and the stability of expected influence (EI) and bridge EI (bEI) [16]. First, the 95% CIs of all edge weights were estimated using nonparametric bootstrapping. Then, the edge weight comparison test was applied to identify edges that exhibited significant differences in size. Second, another bootstrap procedure applying a case-dropping setting was conducted to estimate the stability of centrality indices. The centrality stability-coefficient (CS-coefficient, preferably above 0.50) was determined. The CS-coefficient indicates the largest proportion of samples that could be excluded while still ensuring a 95% probability that the correlation between the original centrality indices remains at or above 0.70 [16,53]. Lastly, a centrality difference test was conducted to determine whether there were any statistically significant differences between the centrality estimates. All bootstraps were set to 5000 times.

#### Network Centrality Indices

The network centrality indices of each network model were calculated using the *centralityPlot* function in the *qgraph* package. EI is mainly reported to represent the cumulative influence of a node within the network in terms of its nature and strength. The EI metric is analogous to the estimation of strength centrality; however, it retains the positive or negative value assigned to the edge weight [54]. The 1-step EI evaluates a node’s impact by considering its immediate neighbors within the network. A bridge node is identified as a node that shares edges with other nodes from different communities (ie, colored differently in the network) in the network.bEI of each node was then estimated within both the comorbidity network and PIU-IES-SC network to identify the most influential bridge nodes [55].

The network comparison test, developed by van Borkulo et al [56], was conducted to assess potential differences in the PIU-IES-SC network characteristics between male and female adolescents with PIU. This analysis was performed using the *NetworkComparisonTest* package and applying false discovery rate correction to all *P* values.

#### Flow Network

To test the potential of *off_SC* as an intervention target, a flow network was generated from the PIU-IES-SC network to identify both the proximal and distal symptoms of *off_SC* (starting from the node *off_SC*).

#### DAG

To further explore the conditional dependencies between PIU symptoms, psychopathological symptoms, and SC, Bayesian network structure learning was used to estimate a DAG using the same set of factor-level variables as in the PIU-IES-SC network. DAG structure learning was performed with the *bnlearn* package in R, using a hill-climbing algorithm as the search procedure. To assess the stability of the resulting structure, we applied a nonparametric bootstrap procedure with 5000 resamples. A consensus DAG was then derived by retaining only those edges that appeared in at least 85% of the bootstrapped networks and edge directions that were supported in more than 50% of the networks [26,27]. The final averaged DAG was visualized with the *Rgraphviz* package. Arrows represent the data-driven direction assignment from structure learning, and edge thickness reflects bootstrap support (edge weight) for the corresponding edge.

#### Sensitivity Analysis

We did 2 additional analyses to ensure the stability of the primary results. First, we repeated the analyses using the more stringent cutoff of ≥70. Moreover, because neglect work and anticipation had α values below 0.70, a second sensitivity analysis re-estimated the network after excluding these 2 factors.

## Results

### Characteristics of Participants

Of 11,946 submitted questionnaires, 2539 were excluded because respondents were outside the target age range (n=509), completed the survey in less than 10 minutes (n=632), selected “false” or “entirely false” for the data-validity item (n=1231), had missing essential demographic information (n=139), or had duplicate responses (n=28). Duplicate responses were identified using identical demographic and school information, with the most complete response retained. Among 9407 valid questionnaires, 2055 (21.8%, 95% CI 21.0%‐22.7%) adolescents (n=1043 males) were identified as having elevated PIU symptoms. The mean age of the overall participants and PIU participants was 14.90 (SD 1.61) years and 15.20 (SD 1.53) years, respectively. Meanwhile, the high-risk and lower-risk groups differed significantly in only-child status, residence, family economic status, and parental education, although the effect sizes were relatively small. The demographic variables and measures of the 2 groups are shown in Table 1.

**Table 1.** Description of study samples and variables^a^.

Measures	Total (N=9407)	High-risk PIU^b^ group (n=2055)	Lower-risk PIU group (n=7352)	Effect size^c^	*P* value
Sex, n (%)				0.025	.02
Male	4556 (48.4)	1043 (50.8)	3513 (47.8)		
Female	4851 (51.6)	1012 (49.2)	3839 (52.2)		
Age (y), mean (SD)	14.90 (1.61)	15.20 (1.53)	14.81 (1.62)	0.25	<.001
Only-child status, n (%)				0.021	.04
No	5173 (55.0)	1090 (53.0)	4083 (55.5)		
Yes	4234 (45.0)	965 (47.0)	3269 (44.5)		
Residence, n (%)				0.067	<.001
Urban	7429 (79.0)	1544 (75.1)	5885 (80.0)		
Town	1264 (13.4)	288 (14.0)	976 (13.3)		
Rural	714 (7.6)	223 (10.9)	491 (6.7)		
Family economic status, n (%)				0.062	<.001
Below average	600 (6.4)	183 (8.9)	417 (5.7)		
Average	6985 (74.3)	1529 (74.4)	5456 (74.2)		
Above average	1822 (19.4)	343 (16.7)	1479 (20.1)		
Father’s education (y), n (%)				0.036	.006
0‐9	3379 (35.9)	756 (36.8)	2623 (35.7)		
10‐12	2390 (25.4)	500 (24.3)	1890 (25.7)		
13‐17	3481 (37.0)	748 (36.4)	2733 (37.2)		
>17	157 (1.7)	51 (2.5)	106 (1.4)		
Mother’s education (y), n (%)				0.035	.01
0‐9	3650 (38.8)	846 (41.2)	2804 (38.1)		
10‐12	2126 (22.6)	424 (20.6)	1702 (23.2)		
13‐17	3545 (37.7)	760 (37.0)	2785 (37.9)		
>17	86 (0.9)	25 (1.2)	61 (0.8)		
*DSM-5-TR^d^* level-2, mean (SD)					<.001
Depression	1.97 (0.94)	2.51 (0.99)	1.82 (0.87)	0.77	
Anxiety	1.92 (0.91)	2.42 (0.95)	1.78 (0.84)	0.74	
Irritability	0.28 (0.43)	0.52 (0.53)	0.21 (0.38)	0.73	
Repetitive T&B^e^	0.49 (0.66)	0.77 (0.78)	0.41 (0.60)	0.55	
BPAQ^f^, mean (SD)					<.001
Anger	2.23 (0.77)	2.81 (0.76)	2.07 (0.70)	1.04	
Physical aggression	2.05 (0.69)	2.52 (0.69)	1.92 (0.63)	0.93	
Verbal aggression	2.55 (0.80)	2.95 (0.69)	2.44 (0.80)	0.67	
Hostility	2.42 (0.86)	3.03 (0.75)	2.25 (0.81)	0.98	
IAT-20^g^, mean (SD)					<.001
Salience	1.85 (0.79)	2.97 (0.65)	1.53 (0.48)	2.76	
Excessive use	1.95 (0.74)	2.98 (0.59)	1.66 (0.46)	2.70	
Neglect work	2.08 (0.82)	3.05 (0.71)	1.81 (0.63)	1.91	
Lack of control	2.2 (0.9)	3.37 (0.66)	1.87 (0.66)	2.27	
Anticipation	1.93 (0.89)	3.07 (0.79)	1.62 (0.62)	2.19	
SCS-R^h^, mean (SD)					<.001
on_SC^i^	3.88 (0.60)	3.80 (0.58)	3.91 (0.60)	−0.18	
off_SC^j^	4.25 (0.80)	3.82 (0.74)	4.37 (0.78)	−0.71	

a High-risk PIU group and lower-risk group were operationally defined as IAT-20 scores of ≥50 and <50, respectively.

b PIU: problematic internet use.

c Effect sizes were calculated using Cramér *V* for categorical variables and Cohen *d* for continuous variables.

d *DSM-5-TR*: *Diagnostic and Statistical Manual of Mental Disorders *(Fifth Edition, Text Revision).

e Repetitive T&B: repetitive thoughts and behaviors.

f BPAQ: Buss-Perry Aggressive Questionnaire.

g IAT-20: Young’s 20-item Internet Addiction Test.

h SCS-R: social connectedness scale-revised.

i on_SC: online social connectedness.

j off_SC: offline social connectedness.

### Undirected Network Analysis

#### Network Robustness

**Figure 1.**
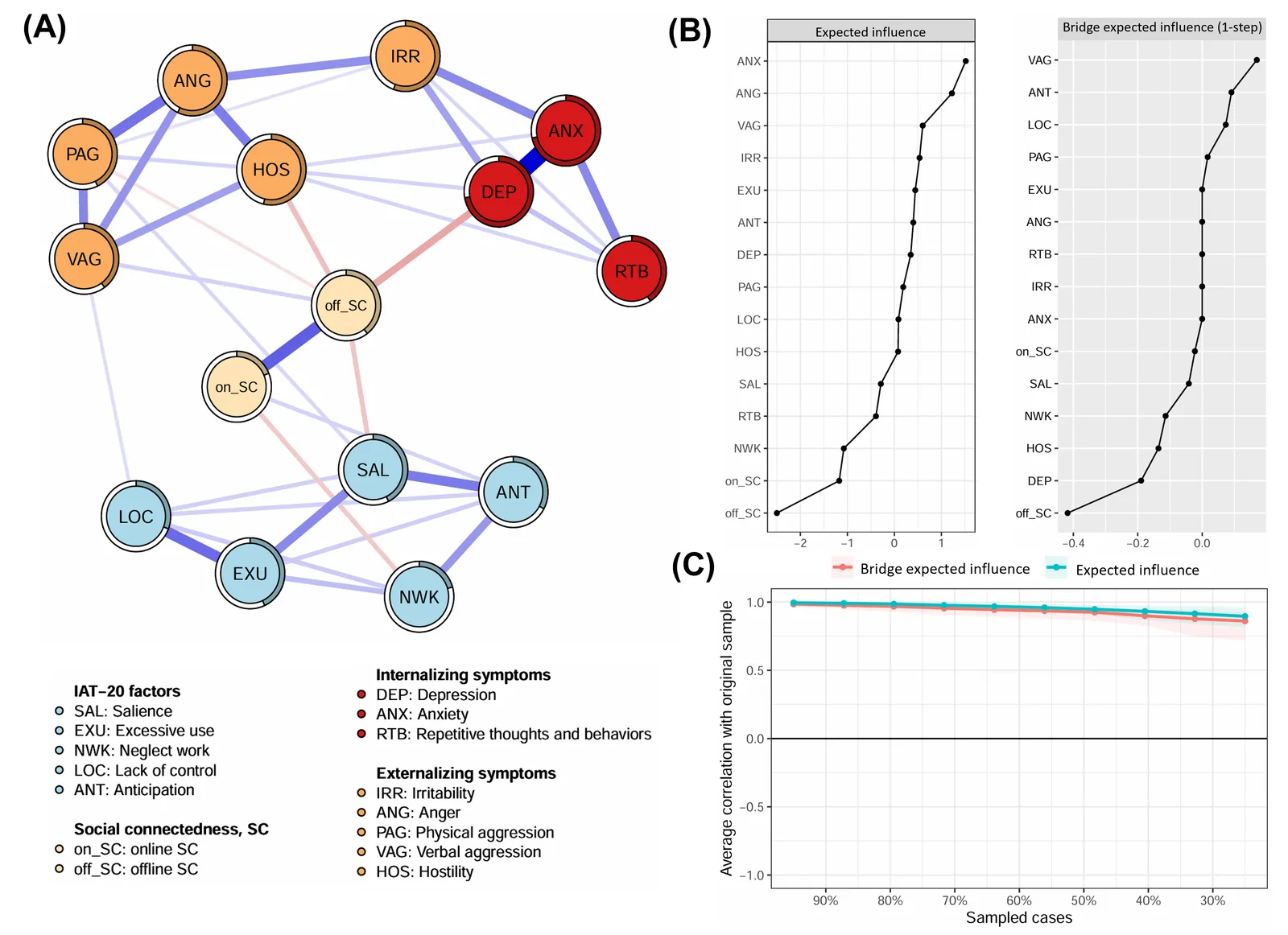
The structure, centrality indices and network stability of the integrative symptom network encompassing PIU, internalizing and externalizing symptoms, and social connectedness (PIU-IES-SC). (A) Model structure of the PIU-IES-SC network. The blue edges represent positive partial correlations, while the red edges represent negative ones. The thickness and saturation of an edge indicate its weight. The ring chart surrounding each node represents the predictability percentage, indicating the extent to which a node shares variance with its neighboring nodes. An abbreviation list for each node is shown in the bottom-left corner. (B) The expected influence (EI, z-score) and bridge expected influence (bEI, 1-step) for each node in the PIU-IES-SC network. The internalizing and externalizing symptoms (colored red and orange in 1A) were identified as a single group in the bEI estimation. (C) Stability of EI and bEI in the PIU-IES-SC network using case-dropping bootstrap. The line chart shows the average correlation between the original sample and the estimated EIs and bEIs during the case-dropping procedure. The blue and red shaded regions represent 95% CIs of EI and bEI, respectively. PIU: problematic internet use; IAT-20: Young’s 20-item Internet Addiction Test.

The bootstrapped 95% CIs for each edge in both the comorbidity and PIU-IES-SC networks are presented in Figures S5 and S10 in Multimedia Appendix 1, respectively. Both demonstrate the high precision of edge weight estimates, as indicated by the narrow 95% CIs. As for the stability of both network models, the CS-coefficient for EI was rather high at 0.75 for both the comorbidity network and the PIU-IES-SC network. However, the CS-coefficient for bEI increased sharply from 0.128 in the comorbidity network to 0.75 in the PIU-IES-SC network (Figure S8 in Multimedia Appendix 1 and Figure 1C, respectively). This suggests greater robustness in the estimation of both node centrality and bridge centrality within the PIU-IES-SC network, compared with the comorbidity network without SC nodes. Additionally, bootstrapped tests of edge weight differences and centrality index differences are illustrated in Figures S6, S7, S11, and S12 in Multimedia Appendix 1, respectively.

#### Network Structure and Centrality of PIU-IES-SC

Figure 1A shows the network structure of the integrative symptom network encompassing IAT-20 factors, IES, and SCs. The mean predictability of all nodes was 0.44 (SD 0.16). The bridge and node centrality of this network are shown in Figure 1B. The key nodes that exhibited the highest positive EI values were *anxiety, anger*, and *irritability* within the network of psychopathology and aggression, playing main roles in the consolidation of clinical symptoms among adolescents with PIU. The node *off_SC* had the most negative edges connected and was identified as the most influential node in disintegrating the network, followed by *on_SC*. Bridge centrality estimation indicated that *off_SC* functioned as the predominant bridge node, exhibiting a negative mediating role among the 3 communities (with 8 psychopathological symptoms treated as a single community). Meanwhile, *depression* also exhibited a negative bEI, primarily due to its strong negative association with *off_SC*. In contrast, *verbal aggression* emerged as another major bridge node, facilitating connectivity among the 3 communities. Other centrality indices are displayed in Figure S9 (Multimedia Appendix 1). Figure S3 in Multimedia Appendix 1 shows the network structure, EI, and bEI of the combined network involving IAT-20 factors and psychopathological symptoms. A description of this network can be found in Multimedia Appendix 1.

Although significant gender distribution differences associated with PIU were revealed in Table 1, the differences in network models between male (n=1043, 50.8%) and female (n=1012, 49.2%) adolescents with PIU were not statistically significant in network global strength (male: 6.48, female: 6.42, *P*=.85, Figures S13 and S14 in Multimedia Appendix 1) or edge weights (M=0.143, *P*=.05)

#### Flow Network Model of off_SC

**Figure 2.**
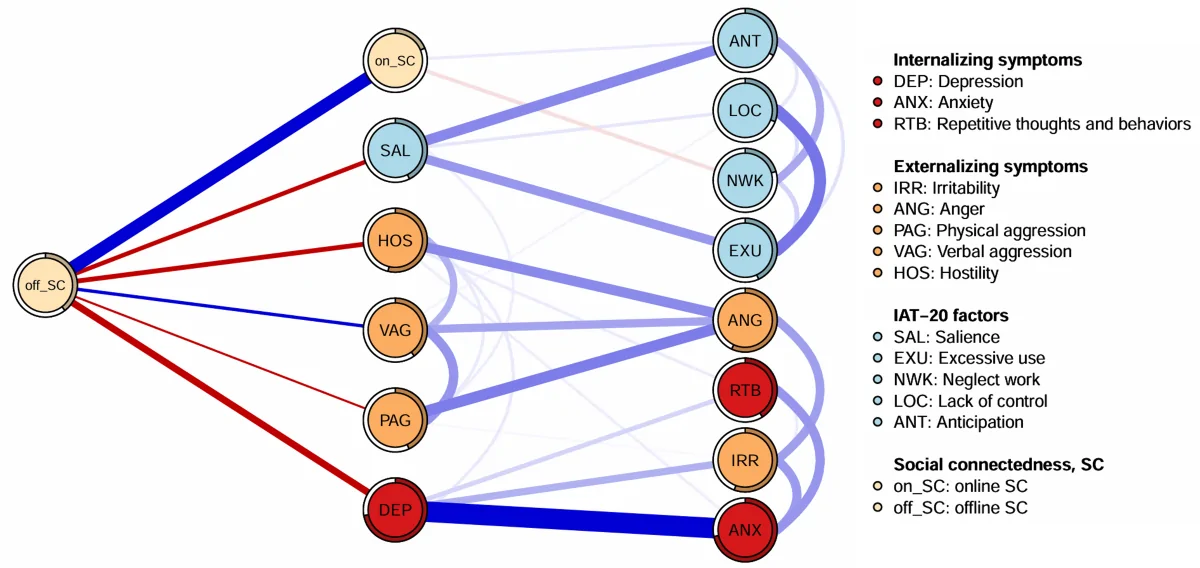
Flow network of offline social connectedness. Nodes directly connected to off_SC are arranged in the left line, while those indirectly associated with off_SC are arranged in the right line. IAT-20: Young’s 20-item Internet Addiction Test. An abbreviation list for each node is shown on the right side.

The flow network of *off_SC*, derived from the PIU-IES-SC network, is shown in Figure 2. *Off_SC* was positively and directly associated with *on_SC* (*r*=0.34) and *verbal aggression* (*r*=0.10). The symptom of *depression*, *salience* of IAT-20 factors, and 2 aggression traits (ie, physical aggression and hostility) were negatively and directly linked to *off_SC*. Among these, *depression* had the strongest correlation with *off_SC* (*r*=−0.19). Other factors and symptoms were indirectly associated with *off_SC* within 2 steps.

### DAG

**Figure 3.**
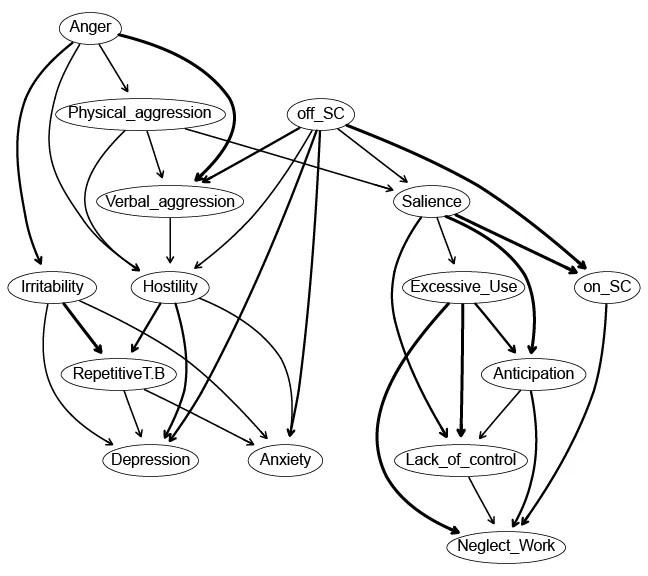
Directed acyclic graph of problematic internet use, internalizing and externalizing symptoms, and social connectedness. Arrows indicate the direction selected by the Bayesian structure-learning algorithm. Edge thickness indicates the bootstrap support for the corresponding edge. off_SC, offline social connectedness; on_SC, online social connectedness; Repetitive T.B: repetitive thoughts and behaviors.

Figure 3 depicts the DAG and the estimated directional dependencies among PIU factors, IES, and SCs in adolescents in the high-risk PIU group. *Off_SC* showed outgoing edges toward externalizing symptoms (*verbal aggression* and *hostility*), internalizing symptoms (*depression* and *anxiety*), *salience* of PIU, and *on_SC*. Externalizing symptoms generally had more outgoing edges within the psychopathology subnetwork, while *anxiety* and *depression* had more incoming edges. Within the PIU symptom subnetwork, *salience* showed outgoing edges toward other PIU factors, whereas *neglect work* primarily received incoming edges. Because multiple DAGs may represent the same conditional independence relationships, these arrow directions cannot indicate any causal effect.

### Sensitivity Analysis

Sensitivity analyses using an IAT-20 cutoff of ≥70 included 287 (3.05%) participants. The principal undirected network was broadly preserved, although bridge CS was low (CS-coefficient≈0.05), and the learned DAG structure differed from the primary analysis. After excluding the *anticipation* and *neglect work* factors, the 13-node network closely resembled the primary network. EI and bEI showed acceptable stability (CS-coefficients≈0.75 and 0.67, respectively). Detailed results are presented in Multimedia Appendix 1.

## Discussion

### Principal Findings

This multicenter study extends previous work by constructing a factor-level symptom network that integrates PIU factors, internalizing and externalizing psychopathological symptoms, and SC in adolescents with a high-risk PIU condition. In the undirected network, both SC variables occupied negative bridge positions, with *off_SC* showing the more prominent role. The exploratory Bayesian network further highlighted the structural prominence of *off_SC*, which showed algorithm-selected outgoing arrows to nodes across the PIU, internalizing, and externalizing domains. *On_SC* occupied a less prominent negative network position and was positively connected with *off_SC*, highlighting the related but distinct positions of SC across the 2 contexts.

This study identified a closely interconnected factor-level structure between PIU symptoms and psychopathological symptoms among adolescents meeting the high-risk PIU threshold, consistent with previous item-level network studies [19,21,22]. Within the comorbidity network, psychopathological symptoms exerted greater influence, suggesting that emotional and behavioral symptoms may be more clinically prominent than PIU-related symptoms among adolescents in the high-risk group. This pattern is consistent with clinical observations that adolescents with PIU may seek treatment for emotional burden or behavioral problems rather than internet overuse alone, and such comorbidities are associated with poorer treatment outcomes [57]. Notably, centrality analyses highlighted *anxiety* as the most influential symptom domain among the psychopathological symptoms. This finding broadly aligns with previous studies identifying anxiety-related symptoms as central or bridging features in PIU-related networks. A recent large-scale study also identified social anxiety as a node with high strength and bridge centrality in the network of PIU and adolescent psychological issues [58].

Complementing this finding, the learned DAG positioned *anger* as a parent node with multiple outgoing arrows, highlighting its prominent structural position in the PIU-psychopathology network. This finding is consistent with longitudinal evidence. A 3-wave study found prospective associations between dysfunctional anger regulation and subsequent internalizing problems [59]. A study of 1103 adolescents also supported the positive association between PIU severity and anger [9]. These findings may help explain why *anger* occupied a prominent cross-domain position in the learned DAG, although its parent-node position should not be interpreted as evidence that *anger* causally precedes the other symptoms.

After incorporating SC factors into the comorbidity network model, we observed a substantial improvement in overall stability, with both the bEI and EI coefficients reaching high levels. This suggests that both *off_SC* and *on_SC* helped compensate for the previously unstable bridging structure and acted as meaningful connectors between PIU and associated psychopathological symptoms. Within the overall PIU-IES-SC network, SC—particularly *off_SC*—appears to play a crucial role in disrupting the reciprocal reinforcement between PIU symptoms and psychopathological symptoms. This is consistent with the study by Prievara et al [60], which also pointed out that social support from offline environments is a protective factor against PIU, and aligns with broader evidence that feeling connected to others is a fundamental human need linked to higher well-being and lower risk of stress disorder, anxiety, and depression [19,61,62]. Generally, offline social activities are more beneficial for mental health than online communication, as face-to-face interactions often provide richer emotional cues, including facial expressions, gestures, and eye contact [63]. Active participation in offline social activities can also reduce adolescents’ screen time, thereby alleviating PIU symptoms [64].

Our flow network model and exploratory DAG provided complementary descriptions of how *off_SC* was embedded within the PIU-IES-SC system. As a reorganization of the undirected network, the flow network showed that all symptom nodes were connected with *off_SC* within 2 steps, indicating that *off_SC* was closely linked to the PIU-psychopathology structure. The exploratory DAG further showed algorithm-selected outgoing arrows from *off_SC* toward several internalizing, externalizing, and PIU-related nodes. Given the cross-sectional design, the observed orientations may reflect *off_SC* as an antecedent, a consequence, or part of a reciprocal process [26]. Relevant longitudinal evidence suggests that the temporal relationship between *off_SC* and PIU may vary according to the type and source of connectedness. In a 3-wave study of 1684 Chinese adolescents, higher school connectedness predicted lower subsequent problematic social network use, and vice versa [65]. In a 4-year study spanning Grades 8 to 11, compulsive internet use predicted later reductions in perceived teacher support; however, parental support predicted subsequent increases in compulsive internet use [66]. Although the causal relationships of *off_SC* with PIU and psychopathological symptoms remain uncertain, its consistently prominent structural position across the PIU-IES-SC network makes it a plausible intervention target. One relevant approach is social prescribing, which links individuals to community-based social resources and activities [67] and may improve social relationships and psychological well-being [68]. For adolescents with elevated PIU symptoms, coordinated family-based, school-based, and community-based programs could promote meaningful offline interaction as an adjunct to established psychological or clinical care.

In the PIU-IES-SC network, *on_SC* also emerged as a protective factor, exhibiting the second-highest negative EI value after *off_SC*. In the digital age, the internet has become a key platform for adolescents to engage socially. Previous evidence has suggested that forming new friendships, exploring personal interests, and sharing stressors on social media help adolescents to develop a sense of identity and help relieve negative emotions such as anxiety and depression [69-71]. *On_SC* was also directly and positively connected with *off_SC*, suggesting that online and offline connectedness may represent related and complementary social resources rather than function as entirely separate domains. In a study of 733 adolescents, those who were highly socially engaged in both contexts reported the most positive self-concepts, whereas those with high online but low offline engagement reported the least positive self-concepts [72]. A longitudinal study similarly found that greater social media use predicted more subsequent time spent with friends offline [73]. These findings suggest that online connectedness may be particularly beneficial when it complements meaningful offline relationships.

Finally, 3 findings warrant cautious interpretation. First, despite the predominantly negative network profile of *off_SC*, it showed a small positive partial association with verbal aggression (*r*=0.10). Because this edge represents the residual association between *off_SC* and verbal aggression after accounting for hostility, physical aggression, depression, and all other network nodes, its small positive value may partly reflect the removal of variance shared with these correlated factors [74]. It is also possible that greater offline engagement increases exposure to interpersonal conflict or peer contexts that reinforce aggressive communication [75]. Given its small magnitude, this association requires replication before substantive conclusions can be drawn. Second, although the proportion of boys and girls differed between the high-risk and lower-risk PIU groups, the network comparison tests detected no significant gender differences in global strength or overall edge structure. This suggests that gender differences in PIU severity or presentation do not necessarily translate into differences in the overall pattern of associations among PIU, psychopathological symptoms, and SC. Previous studies have also reported mixed findings. One longitudinal PIU network study reported similar network structures and global strength across boys and girls [76], whereas another large-scale adolescent study identified gender-specific PIU network characteristics [25]. Thus, the present findings indicate that no clear gender difference was detected in this sample. Third, when the stricter IAT-20 cutoff of ≥70 was applied, the overall undirected network was broadly preserved, but bridge centrality showed low stability and the learned DAG differed from that obtained in the primary analysis. One likely explanation is the substantial reduction in sample size, from 2055 participants in the primary analysis to only 287 under the stricter cutoff. Nevertheless, the discrepancy may also reflect genuine differences in the dependency structure of adolescents with more severe PIU symptoms. Therefore, the bridge-node rankings and algorithm-selected DAG orientations under different PIU thresholds should be regarded as exploratory and require replication in larger samples with severe PIU.

### Limitations

Despite the insights provided by this study, several limitations should be acknowledged. First, the cross-sectional nature of the data precludes definitive conclusions about causality, and our sampling logic of schools may limit the generalizability of the findings. While our findings allow for potential causal hypotheses based on partial correlation networks and prior knowledge, longitudinal studies using more representative samples are needed to further validate these assumptions. Second, the adapted SCS-R scales have not been independently validated before; therefore, the online-offline differences and their network positions should be interpreted cautiously. Third, several potentially relevant factors, including family functioning, socioeconomic status, and personality traits, were not included in the network models. These factors may be associated with both SC and psychopathological symptoms, and their omission may have influenced the estimated network structure and DAG orientations. Finally, the internalizing and externalizing psychopathological symptoms included in this study were limited to those commonly associated with PIU. Other co-occurring symptoms, such as insomnia and psychotic-like experiences, were not included, which may have constrained the comprehensiveness of the symptom network.

### Conclusions

This study characterized a closely interconnected factor-level network linking PIU with internalizing and externalizing psychopathological symptoms among adolescents with high-risk PIU symptoms. *Off_SC* showed the most prominent negative bridge position and was directly or indirectly connected with all network nodes within 2 steps. *On_SC* showed a secondary negative network position and was positively associated with *off_SC*, suggesting that connectedness across the 2 contexts may represent related social resources. These findings identify *off_SC* as a plausible candidate for further prevention and intervention research. Longitudinal and interventional studies using validated context-specific measures are needed to examine directionality and determine whether SC-focused strategies can improve PIU-related and mental health outcomes.

## Supplementary material

10.2196/92473 Multimedia Appendix 1 Supplementary network structures, expected influence, centrality indices, bootstrap stability analyses, gender comparisons, and sensitivity analyses of the psychopathological symptom, problematic internet use, and comorbidity networks.

## Abbreviations

bEI: bridge expected influence
CS-coefficient: centrality stability-coefficient
DAG: directed acyclic graph
*DSM-5-TR*: *Diagnostic and Statistical Manual of Mental Disorders* (Fifth Edition, Text Revision)
EI: expected influence
IAT-20: 20-item Internet Addiction Test
IES: internalizing/externalizing symptoms
off_SC: offline SC
on_SC: online SC
PIU: problematic internet use
PIU-IES-SC: PIU-internalizing/externalizing symptoms-social connectedness network
SC: social connectedness
SCS-R: Social Connectedness Scale-Revised
