# Supplementary material for: Social Connectedness Buffering the Interaction Between Problematic Internet Use and Psychopathological Symptoms Among Adolescents: Undirected and Bayesian Network Analyses
Source: J Med Internet Res. 2026 Aug 31;28:e92473. doi: 10.2196/92473 (PMC13528827; doi:10.2196/92473)
Supplement: Supplementary file 1 [file jmir-v28-e92473-s001.docx]

**Supplementary Materials**

[**Table S1.** The list of node numbers and abbreviations in this study.	2](#_Toc235780284)

[**Figure S1**. Network structure and expected influence of the psychopathological symptom network	2](#_Toc235780285)

[**Figure S2.** Network structure and expected influence of the PIU network.	3](#_Toc235780286)

[**Figure S3.** The structure and centrality indices of the comorbidity network involving PIU and internalizing and externalizing symptoms.	4](#_Toc235780287)

[**Figure S4.** Centrality indices of the comorbidity network.	5](#_Toc235780288)

[**Figure S5.** Bootstrapped confidence intervals of edge weights in the comorbidity network	6](#_Toc235780289)

[**Figure S6.** Estimation of edge weight difference in the comorbidity network by bootstrapped difference test	7](#_Toc235780290)

[**Figure S7.** Estimation of centrality difference of nodes in the comorbidity network by bootstrapped difference test	8](#_Toc235780291)

[**Figure S8.** Stability of expected and bridge expected influence in comorbidity network using case-dropping bootstrap	9](#_Toc235780292)

[**Figure S9.** Centrality indices of the PIU-IES network.	10](#_Toc235780293)

[**Figure S10.** Bootstrapped confidence intervals of edge weights in PIU-IES network	11](#_Toc235780294)

[**Figure S11.** Estimation of edge weight difference in PIU-IES network by bootstrapped difference test	12](#_Toc235780295)

[**Figure S12.** Estimation of centrality difference of nodes in PIU-IES network by bootstrapped difference test	13](#_Toc235780296)

[**Figure S13.** Network structure of PIU, psychopathological symptoms and SCs in males and females	14](#_Toc235780297)

[**Figure S14**. Comparison of network properties between males and females	15](#_Toc235780298)

[**Sensitivity Analysis**	16](#_Toc235780299)

[**Figure S15.** Estimated PIU-IES network among adolescents with IAT-20 scores ≥70 (n=287).	18](#_Toc235780300)

[**Figure S16.** Expected influence in the PIU-IES network using the IAT-20 cutoff of ≥70.	19](#_Toc235780301)

[**Figure S18.** Bootstrapped directed acyclic graph using the IAT-20 cutoff of ≥70.	21](#_Toc235780302)

[**Figure S19.** Estimated 13-node network after excluding the anticipation and neglect-work factors (n=2055).	22](#_Toc235780303)

[**Figure S20.** Expected influence in the 13-node network after excluding the anticipation and neglect-work factors.	23](#_Toc235780304)

[**Figure S21.** Raw bridge expected influence in the 13-node network after excluding the anticipation and neglect-work factors.	24](#_Toc235780305)

[**Figure S22.** Bootstrapped directed acyclic graph after excluding the anticipation and neglect-work factors.	25](#_Toc235780306)

**Table S1.** The list of node numbers and abbreviations in this study.

| **Number** | **Node name** | **Abbreviation** |
| --- | --- | --- |
| 1 | Depression | DEP |
| 2 | Anxiety | ANX |
| 3 | Irritability | IRR |
| 4 | Repetitive Thoughts and Behaviors | RTB |
| 5 | Anger | ANG |
| 6 | Physical aggression | PAG |
| 7 | Verbal aggression | VAG |
| 8 | Hostility | HOS |
| 9 | Salience | SAL |
| 10 | Excessive use | EXU |
| 11 | Neglect work | NWK |
| 12 | Lack of control | LOC |
| 13 | Anticipation | ANT |
| 14 | Offline social connectedness | off_SC |
| 15 | Online social connectedness | on_SC |

**Figure S1**. Network structure and expected influence of the psychopathological symptom network

*Note. The expected influence (z-score) and bridge expected influence (effect size) of each node is displayed in line graphs. The blue edges represent positive correlations, while the red edges represent negative ones. The thickness and saturation of an edge indicate its weight. The ring chart surrounding each node represents the predictability percentage, indicating the extent to which a node shares variance with its neighboring nodes.*

**Figure S2.** Network structure and expected influence of the PIU network.

*Note. The expected influence (z-score) and bridge expected influence (effect size) of each node is displayed in line graphs. The blue edges represent positive correlations. The thickness and saturation of an edge indicate its weight. The ring chart surrounding each node represents the predictability percentage, indicating the extent to which a node shares variance with its neighboring nodes.*

**Figure S3.** The structure and centrality indices of the comorbidity network involving PIU and internalizing and externalizing symptoms.

Note: PIU, problematic Internet use; IAT-20, Young’s 20-item Internet Addiction Test. The expected influence (z-score) and bridge expected influence (effect size) of each node is displayed in line graphs. The internalizing and externalizing symptoms (colored red and orange) were identified a one single group in the bEI estimation. The blue edges represent positive correlations, while the red edges represent negative ones. The thickness and saturation of an edge indicate its weight. The ring chart surrounding each node represents the predictability percentage, indicating the extent to which a node shares variance with its neighboring nodes. Abbreviation list for each node was shown on the right side.

The mean predictability of all nodes was 0.46. In this comorbidity network, *anxiety*, *anger* and *depression* were identified as the most influential nodes. Nodes within the domain of psychopathological symptoms showed strong interconnectivity and formed tight communities. Thus, the eight nodes highlighted in yellow and red in Figures 1 and 2 were treated as one community for assessing bridge centrality indices. *Salience,* *verbal aggression* and *depression* served as primary bridge nodes within the network. Between two communities, *physical aggression* and *lack of control* were negatively correlated with each other (r = -0.08). Other centrality indices (strength, closeness and betweenness) are displayed in Figure S3. The strongest correlations were found between *depression* and *anxiety* (r = 0.56); *anger* and *physical aggression* (r = 0.30); *excessive use* and *lack of control* (r = 0.27). The structure and EI indices for both the independent PIU network model and the psychopathological symptom network model are presented in the supplementary material.

**Figure S4.** Centrality indices of the comorbidity network.

*Note. The corresponding node names can be found in Supplementary Table S1*

**Figure S5.** Bootstrapped confidence intervals of edge weights in the comorbidity network

*Note. The gray area represents the estimated 95% confidence intervals (CIs) for each edge weight. The corresponding node names can be found in Supplementary Table S1.*

**Figure S6.** Estimation of edge weight difference in the comorbidity network by bootstrapped difference test

*Note. Edges that differ significantly from each other in weight are represented in black boxes. The corresponding node names can be found in Supplementary Table S1.*

**Figure S7.** Estimation of centrality difference of nodes in the comorbidity network by bootstrapped difference test

*Note. Nodes differing in expected influence, i.e. the sum of all edge weights connected to a given node. Significant differences are highlighted by black boxes, while the strength values are indicated along the diagonal. The corresponding node names can be found in Supplementary Table S1.*

**Figure S8.** Stability of expected and bridge expected influence in comorbidity network using case-dropping bootstrap

*Note. The average correlation between the original sample and the estimated EIs and bEIs during the case-dropping procedure. The blue and red shaded regions represent 95% CIs of EI and bEI, respectively.*

**Figure S9.** Centrality indices of the PIU-IES network.

*Note. The corresponding node names can be found in Supplementary Table S1*

**Figure S10.** Bootstrapped confidence intervals of edge weights in PIU-IES network

*Note. The gray area represents the estimated 95% CIs for each edge weight. The corresponding node names can be found in Supplementary Table S1.*

**Figure S11.** Estimation of edge weight difference in PIU-IES network by bootstrapped difference test

*Note. Edges that differ significantly from each other in weight are represented in black boxes.*

**Figure S12.** Estimation of centrality difference of nodes in PIU-IES network by bootstrapped difference test

*Note. Nodes differing in expected influence, i.e. the sum of all edge weights connected to a given node. Significant differences are highlighted by black boxes, while the strength values are indicated along the diagonal.*

**Figure S13.** Network structure of PIU, psychopathological symptoms and SCs in males and females

*Note. The blue edges represent positive correlations, while the red edges represent negative ones. The thickness and saturation of an edge indicate its weight. Corresponding node names are shown on the right side.*

**Figure S14**. Comparison of network properties between males and females

Note. **Left panel:** Bootstrapped difference in global network strength based on 1,000 permutations; the difference between male and female adolescents with PIU was not significant (male = 6.48, female = 6.42; S = 0.068, *p* = 0.853). **Right panel:** Bootstrapped maximum difference in edge weights based on 1,000 permutations; the difference was also not significant (M = 0.143, *p* = 0.053).

**Sensitivity Analysis**

**Sensitivity analysis 1: IAT-20 cut-off ≥70**

All network analyses were repeated among adolescents with IAT-20 scores ≥70. This more stringent threshold identified 287 of 9407 participants (3.05%). The same 15 nodes, community assignments, network-estimation procedures, bootstrap procedures, and DAG procedures as in the primary analysis were used.

**Main results:**

The strongest EI values were observed for anger (1.087), anxiety (1.039), and depression (0.988). Offline social connectedness retained negative bridge expected influence at both one step (−0.152) and two steps (−0.318); its two-step value was the lowest among all nodes. However, the correlation-stability coefficient was 0.596 for expected influence but only 0.049 for bridge expected influence, indicating that the bridge-centrality estimates were unstable in this smaller subsample.

In the bootstrapped DAG, offline social connectedness was linked to depression and online social connectedness. The retained directions were depression → offline social connectedness (edge inclusion strength=0.909; direction probability=0.842) and offline → online social connectedness (strength=0.937; direction probability=0.632). Thus, the undirected pattern remained broadly interpretable, whereas the directed position of offline social connectedness differed from the primary DAG and should be interpreted cautiously.

**Sensitivity analysis 2: Exclusion of two lower-reliability IAT-20 factors**

Among adolescents meeting the primary IAT-20 cutoff of ≥50 (n=2055), the anticipation and neglect-work factors were removed and the analyses were repeated using the remaining 13 nodes. Edge weights and EI rankings for nodes shared by the 15-node and 13-node networks were compared using Pearson and Spearman correlations, respectively.

**Main results:**

**Sensitivity analysis 2: 13-node network**

The reduced network closely reproduced the primary 15-node network. Across shared edges, Pearson and Spearman correlations of edge weights were 0.997 and 0.999, respectively; the Spearman correlation between EI rankings was 0.951. Anxiety (1.121), anger (1.034), and verbal aggression (0.848) had the highest expected influence. Offline social connectedness had the most negative one-step (−0.418) and two-step (−0.758) bridge expected influence. Stability was good for both expected influence (CS=0.750) and bridge expected influence (CS=0.672).

The reduced-node DAG retained outgoing edges from offline social connectedness to verbal aggression, hostility, PIU salience, and online social connectedness, while depression preceded offline social connectedness. These findings support the robustness of the undirected network results but also indicate some sensitivity of DAG orientations to node specification.

**Summary of sensitivity analyses**

| **Analysis** | **Sample/nodes** | **Agreement or central findings** | **CS coefficients** | **Interpretation** |
| --- | --- | --- | --- | --- |
| IAT-20 ≥70 | n=287; 15 nodes | Anger, anxiety, and depression had the highest EI; off_SC had the lowest two-step bEI | EI=0.596; bEI=0.049 | Directionally similar bridge finding, but bEI was unstable |
| Removed anticipation and neglect work | n=2055; 13 nodes | Edge weights: Pearson r=0.997, Spearman r=0.999; EI ranks: Spearman ρ=0.951 | EI=0.750; bEI=0.672 | Undirected network findings were highly robust |

**Table S1.** Summary of the two sensitivity analyses. CS, correlation-stability coefficient; EI, expected influence; bEI, bridge expected influence; off_SC, offline social connectedness.

**Figure S15.** Estimated PIU-IES network among adolescents with IAT-20 scores ≥70 (n=287).

Blue edges indicate positive associations and red edges indicate negative associations; thicker edges represent stronger regularized partial correlations.

**Figure S16.** Expected influence in the PIU-IES network using the IAT-20 cutoff of ≥70.

**Figure S17.** Raw bridge expected influence in the PIU-IES network using the IAT-20 cutoff of ≥70.

**Figure S18.** Bootstrapped directed acyclic graph using the IAT-20 cutoff of ≥70.

Edge width represents inclusion strength and arrow direction represents the more frequently observed orientation among retained edges.

**Figure S19.** Estimated 13-node network after excluding the anticipation and neglect-work factors (n=2055).

**Figure S20.** Expected influence in the 13-node network after excluding the anticipation and neglect-work factors.

**Figure S21.** Raw bridge expected influence in the 13-node network after excluding the anticipation and neglect-work factors.

**Figure S22.** Bootstrapped directed acyclic graph after excluding the anticipation and neglect-work factors.

Abbreviations: ANX, anxiety; ANT, anticipation; bEI, bridge expected influence; CS, correlation-stability coefficient; DAG, directed acyclic graph; DEP, depression; EI, expected influence; IAT-20, Young’s 20-item Internet Addiction Test; NWK, neglect work; off_SC, offline social connectedness; on_SC, online social connectedness; PIU, problematic internet use.
